# Personal

**Published:** 1893-09

**Authors:** 


### Personal.

Dr. Nelson G. Richmond, of Fredonia, was elected president of the medical society of the county of Chautauqua at its annual meeting, held at the Chautauqua House, Mayville, N. Y., on Wednesday, July 12, 1893. This is a fitting recognition of the talent and executive ability of one of the most prominent members of the society.

Dr. Richmond and Dr. H. J. Dean, of Brocton, were chosen delegates from the medical society of the county of Chautauqua to the medical society of the State of New York for three years.

